# Towards Measuring Terahertz Photon Statistics by a Superconducting Bolometer

**DOI:** 10.3390/s21154964

**Published:** 2021-07-21

**Authors:** Pavel Prudkovskii, Andrey Leontyev, Kirill Kuznetsov, Galiya Kitaeva

**Affiliations:** Faculty of Physics, Lomonosov Moscow State University, 119991 Moscow, Russia; vysogota@gmail.com (P.P.); aa.leontjev@physics.msu.ru (A.L.); kirill_kuznetsov@physics.msu.ru (K.K.)

**Keywords:** spontaneous parametric down-conversion, superconducting bolometer, photon statistics

## Abstract

Statistical distributions of the analog readings of an antenna-coupled THz superconducting bolometer were measured and analyzed under a special type of irradiation by low-energy fluxes of THz photons with Poisson photon statistics and controllable mean photon numbers. The photons were generated via low-gain parametric down-conversion in pulse-pumped Mg:LiNbO_3_ crystal placed to a cooled cryostat together with the bolometer NbN film. Results of theoretical approximation of experimental histograms reveal the discrete nature of THz detection by superconducting bolometers and open a way for studying their quantum characteristics. It is shown that bolometer readings per pulse consist of discrete counts (“single charges”), with the mean number linearly dependent on the number of input photons. Contributions of single counts to a total analog reading are statistically distributed according to the normal law, with average values slightly depending on the number of counts in each reading. A general formula is proposed to describe the relationship between continuous statistical distribution of the bolometer readings and discrete quantum statistics of the incident photons.

## 1. Introduction

Nowadays, a new direction in terahertz science begins, aimed to extend quantum optical technologies to the terahertz frequency range (THz) [1,2,3,4,5,6,7,8,9,10,11]. Currently developed for the optical range, the methods of quantum field sensing [12,13,14], imaging [15,16], spectroscopy [17,18], and calibration [19,20] potentially can have great prospects in THz. Moreover, studying the statistical parameters of THz radiation at the photon level could give new insights on specific quantum THz states and their interaction with matter. However, up to now all the experimental progress within this direction is concerned with detection of optical signal radiation which is correlated with its THz idler counterpart. It takes place in sensing/spectroscopy applications of quantum-correlated optical-terahertz biphotons generated under spontaneous parametric down-conversion (PDC) [6,8,9,11,21], and in studying the vacuum fluctuations at THz frequencies by means of electro-optic sampling [2,7]. One of the limiting reasons is the great difficulty in creating THz receivers that can operate in the single-photon detection mode [22,23,24,25] and potentially allow the use of high-speed coincidence schemes. It is also an unsolved problem to construct a photon number resolving detector [26,27,28] in the THz range, since the photocurrent shifts related to detection of an additional THz photon are usually too small [29].

Although THz detectors developed to date are still used exclusively in the analog data output mode, some of them have sufficiently high sensitivity and low levels of intrinsic electronic noise. They could be tested by detecting low-photon fluxes, but usually the fluxes are too high due to the thermal background radiation at room temperatures. In our work, we study the capabilities of this type of detectors, namely, superconducting hot-electron bolometers (HEB) [30,31,32,33], to find the way for connecting statistical distributions of their analog signal with statistics of the input THz photons. A necessary and important point of this research was a special creation of a THz source with low and easy-controllable mean number of THz photons per mode. THz photons were generated in a nonlinear crystal as a result of low-gain parametric down-conversion of pulsed laser radiation. Together with the sensitive element of the bolometer and focusing elements of the terahertz path, the crystal was placed in a cooled helium cryostat, which was closed from thermal radiation from external sources.

Histograms of statistical distributions of superconducting NbN bolometer readings, measured over the times of individual laser pulses at different levels of pump powers, were analyzed. Using several commonsense assumptions, an approximation formula has been proposed that fits these distributions with rather high accuracy. It is shown that bolometer readings consist of discrete single counts, and the mean numbers of such counts are dependent on the number of input photons. The linear dependence with nearly the same quantum efficiency was observed at the levels of detected counts up to ~15–20 counts per pulse. It has been examined during the fitting procedures that namely high dispersion of statistical distribution of the single-count contributions to the total reading does not allow direct bolometer operating in the photon-counting mode. However, our analysis made it possible to finally formulate an analogue of Mandel formula which connects continuous statistical distribution of the bolometer readings with discrete quantum statistics of the incident photons.

## 2. Materials and Methods

**Figure 1:**
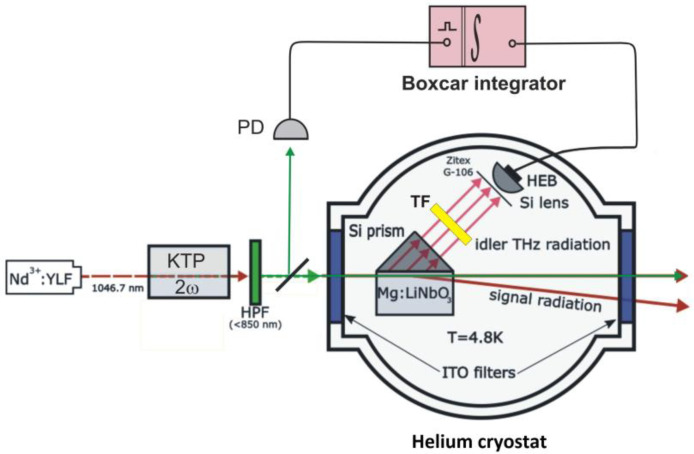
Schematics of the experimental set-up. The pumping radiation for PDC is generated using a series of the pulsed laser (Nd^3+^:YLF), the frequency doubling crystal (KTP), and the 1st harmonic cut-off filter (HPF). A small part of the pump beam is diverted by a mirror to a PIN diode (PD) which triggers integrator and boxcar averager modules SR250 (Boxcar integrator) at the beginning of each laser pulse. PDC originates in Mg-doped lithium niobate crystal (Mg:LiNbO_3_) equipped by a prism from high-resistivity silicon (Si prism). The crystal and a system for detecting idler THz radiation are placed inside a He cryostat. The detection system contains a pump cut-off filter film (Zitex G-106), a narrow THz band pass filter Tydex BPF1.0-24/31 (TF), a focusing Si hemispherical lens (Si lens), and the NbN superconducting bolometer SCONTEL (HEB). The optical photons (signal radiation) are generated at small angles to the pump beam and leave the cryostat via the same windows as the pump radiation passes (marked by the blue color). In each window there is an external glass filter and an internal ITO filter. The latter is kept at the same temperature as the crystal and HEB.

The schematics of our experimental setup are shown in Figure 1. Second harmonic of diode-pumped Nd^3+^:YLF solid-state laser, generated in KTP crystal (2ω), was used to pump Mg:LiNbO_3_ crystal with 7.1 mol.% Mg content (LN). The pump wavelength was 523.35 nm, the pump pulse duration and repetition frequency were 10 ns and 7 kHz, correspondingly, and the pump beam waist at the crystal was ~100 μm. The crystal was mounted in He cryostat together with superconducting HEB manufactured by company SCONTEL, Russia [32]. Temperatures of the crystal and the bolometer sensitive NbN element were kept at the same value: 4.8 K. According to data provided by manufacturers, the characteristic response time and noise equivalent power of the bolometer were 50 ps and $2.5\;\times {\;10}^{-13}W\cdot {\mathrm{Hz}}^{-1/2}$, respectively. A special planar logarithmic spiral antenna [33] was optimized for the spectral range 0.1–6 THz. The cryostat windows of 20 mm diameter, designed to enter the pump radiation into the cooled chamber and to extract it out after passing the crystal, were equipped with ITO filters [34]. The bolometer was inside three shielding shells of the cryostat. Glass windows were installed on an external aluminum shell that was kept at room temperature. The next shell from a stainless steel was provided by open windows. Finally, a third brass shell was in contact with the cooled board. The ITO filters were installed into the windows in the third shell, and the bolometer sensitive element was attached to the board. All cryostat compartments were pumped out to a pressure of 10^−6^ mbar. By this arrangement, the temperature of both ITO filters practically coincided with the temperature of the bolometer, and these cooled filters prevented outside thermal radiation at THz frequencies from entering the cryostat [5]. At the same time, not only direct pumping beam, but also optical radiation of Stokes and anti-Stokes frequencies, generated in LN at small angles, could leave the cryostat without any significant absorption [11]. In addition, IR filter (ZitexRG-106) protected the bolometer from pump radiation elastically scattered within the crystal.

Parametric down-conversion in bulk 0.9 cm Mg:LiNbO_3_ crystal was excited in type-0 geometry. According to phase-matching conditions, the photons of ~1 THz frequency were most effectively generated in this sample at polar angles $\theta_{i0}\approx 60^{\circ }$ with respect to pump direction [4,10]. THz part of PDC radiation was extracted from the crystal through its side face in immediate proximity to the pump beam [5]. A high-resistivity silicon 45° prism was used in order to avoid total internal reflection of THz waves at the output crystal surface. After passing the prism, a part of PDC radiation was selected by a band pass filter (Tydex BPF1.0-24/31) and focused by 5 mm Si hemispherical lens to the bolometer input window. The total spectrum of terahertz photons which can be generated under PDC in the sideways geometry with Mg:LiNbO_3_ crystal occupies the range from 0.1 THz up to 7.2 THz [35]. A narrow band pass filter with a central frequency of 1 THz and a half-width of 0.15 THz transmitted a part of this wide spectrum. The energy bandgap in NbN is 4.5 meV [36], which corresponds to a frequency of 1.15 THz. However, as it was shown experimentally in [33], the spectral responsivity of our bolometer is the highest in the range 1–2.5 THz. So, the spectral range of THz photons incident on the bolometer in our set-up hits the range of its maximal responsivity. Electric signal from HEB detector was amplified by a broadband cryogenic amplifier associated with the bolometer and then directed to time-gated Boxcar integrator SRS 250. The integrator was gated by pulses from a pin-diode (PD) placed in the lateral pump beam outlet, the integration strobe window was 6 ns.

## 3. Results and Discussion

According to general theory of PDC [37], the total mean number of PDC-generated and incident on the bolometer THz photons per second depends on laser pump intensity $I_{p}$ via the parametric gain coefficient β as

(1) $$\langle N\rangle =\frac{{\omega i}^{2}\Delta \omega_{i}\Delta \Omega_{i}S}{{\left(2\pi \right)}^{3}c^{2}}(1+\langle N_{T}\rangle )\cdot \sinh^{2}(\beta ).$$

Here, $\beta =\frac{8d_{33}L\sqrt{\pi^{3}\omega_{s}\omega_{i}}}{\sqrt{c^{3}n_{p}n_{s}n_{i}\cos\theta_{s0}\cos\theta_{i0}}}\sqrt{I_{p}}$, *d*_33_ is the crystal nonlinear coefficient, L is its length, $\Delta \omega_{i}$, $\Delta \Omega_{i}$, and S are the spectral band, a solid angle, and an input area of the detection system, correspondingly. Planck’s factor $\langle N_{T}\rangle ={\left[\exp(\hbar \omega_{i}/k_{B}T)-1\right]}^{-1}$ accounts thermal field fluctuations at the crystal temperature T. Frequencies of quantum-correlated THz ($\omega_{i}$) and optical ($\omega_{s}$) photons are related with the pump frequency $\omega_{p}$ via the energy conversation law, $\hbar \omega_{s}+\hbar \omega_{i}=\hbar \omega_{p}$; $n_{p},n_{s},n_{i}$ are the crystal refractive indexes at corresponding frequencies, $\theta_{s0},\theta_{i0}$ are the propagation angles of optical and THz photons generated under exact phase-matching condition. Evidently, the mean THz wave power and, correspondingly, the averaged HEB readings should demonstrate the same dependence on pump power as is predicted by Equation (1). It was shown in our previous study of PDC process for the same LN crystal sample [38] that the numerical relation

(2) $$\beta =0.1\sqrt{P_{pump}[\mathrm{mW}]}$$

fits well with approximation of the averaged bolometer reading dependence on pump power $P_{pump}$ in milliwatts.

In the present work we record statistical distribution of bolometer short-time readings taken at each pump pulse. A value of an electric charge (in arbitrary units) acquired during each strobe time was issued in the analog form at the Boxcar output. The Boxcar readings were obtained equal to this charge normalized by the strobe time τ=6 ns, which corresponded to the mean photo-induced HEB current at the strobe period. Communication between Boxcar and PC was organized using high bit rate ADC converter. Specially developed software was used to select the photo-induced readings from the background noise, which was received without reference to stroboscopic pulses.

**Figure 2:**
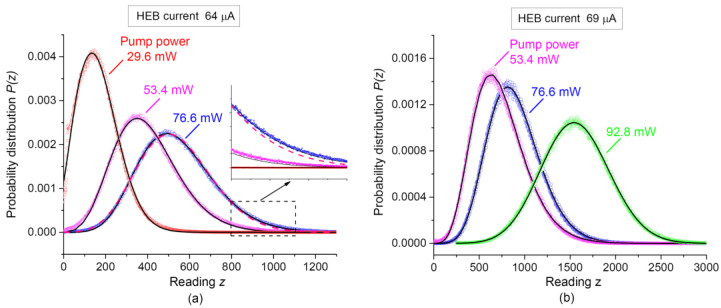
Histograms of the HEB detector readings, recorded experimentally under different laser pump powers (circles), and results of their numerical modeling (curves). The time-averaged pump power was 29.6 mW (red circles), 53.4 mW (magenta circles), 76.6 mW (blue circles), and 92.8 mW (green circles). The transport current of HEB was 64 µA (a) and 69 μA (**b**). Solid curves: numerical approximation by Equation (7). A dashed curve (**a**): approximation by Equation (6); the inset shows a plot section in an enlarged scale.

Examples of area-normalized histograms of the bolometer short-time readings, recorded under different laser pump powers, are shown in Figure 2 by circles. The bolometer operated at two different initial transport currents I through NbN film, 64 μA (Figure 2a), or 69 μA (Figure 2b). The HEB settings, as well as all parameters of PDC apart from pump power, were kept constant within each series of histograms.

Our modeling of observed statistical distributions proceeds from a general assumption that events of photon absorption by an intensity-sensitive detector lead to formation of short-time photocurrent surges at the detector’s output. In case of the superconducting bolometer, this single surge is originated by a local switching from the superconducting or resistive (bimodal superconducting) states in NbN film. When the photon frequency is comparable with the superconducting gap frequency of HEB microbridge, formation of the single current surge can occur in a number of different ways [39,40,41]. This leads to a large spread of amplitudes and temporal distributions of such elementary photocurrent pulses. As a result, a corresponding individual charge contribution $e_{1}$ to the total charge recorded at Boxcar output during the strobe time can take on different values. It is natural to assume that statistical probability distribution $P(e_{1})$ of possible single charge obeys the normal Gaussian law

(3) $$P(e_{1})=\frac{1}{\tilde{\sigma }\sqrt{2\pi }}e^{-\frac{{\left(e_{1}-\langle e_{1}\rangle \right)}^{2}}{2{\tilde{\sigma }}^{2}}}$$

with some mean single charge $\langle e_{1}\rangle$ and dispersion ${\tilde{\sigma }}^{2}$.

Here, by a “single charge” we mean the charge that appears at the output during a separate act of the resistive transition in a superconducting film. Regardless of whether more than one photon is absorbed during the time of this elementary resistive pulse, the single charge corresponds to an individual single count. However, a number m>1 of elementary resistive transitions can proceed during an acquisition time τ (6 ns in our set-up) and the total charge detected by Boxcar presents the sum of all recorded single charges. Let us first assume that this does not affect parameters of the single charge distribution in Equation (3). In this case, a random value of the total output current $z=mi_{1}$ (here, $i_{1}\equiv e_{1}/\tau$ is an elementary photocurrent) should be described by a normal distribution $P_{c}(z|m)$ with the mean value and dispersion being equal to sums of corresponding single-photon parameters, $\langle z\rangle =m\langle e_{1}\rangle /\tau$ and $m\sigma^{2}=m{\left(\tilde{\sigma }/\tau \right)}^{2}$:

(4) $$P_{c}(z|m)=\frac{1}{\sigma \sqrt{2\pi m}}e^{-\frac{{\left(z-m\langle e_{1}\rangle /\tau \right)}^{2}}{2m\sigma^{2}}}.$$

Finally, to predict probability distribution of the detector readings P(z), one has to determine the convolution of P(z|m) with the distribution of photo-counts,

$$P(z)=\sum_{m=1}^{\infty }P(z|m)P(m).$$

Statistical distribution of photo-counts is determined by the photon probability distribution P(N) in the input radiation [42]. In each separate mode of signal or idler radiation, the PDC radiation has a thermal-type (Gaussian) statistics [37]. However, if a large number of modes are detected in each channel (as in our low-frequency “idler” terahertz channel), then, within a semi-classical approach, the intensity of the detected field is considered as constant, and due to the presence of shot noise, the statistics of photo counts has the Poisson character. Alternatively, when describing both the field and the detection process within a completely quantum theory [43], the Poisson statistics are also obtained for the detected photo counts. Therefore, these two different descriptions of the same physical situation predict the same distribution of the photo counts

(5) $$P(m)=\frac{e^{-\langle m\rangle }{\langle m\rangle }^{m}}{m!}.$$

The mean number of photo-counts 〈m〉 should depend on the bolometer quantum efficiency η and an average number of the input photons 〈N〉 as 〈m〉=η〈N〉. Thus, taking into account Equation (4), the distribution of detector readings z can be depicted as

(6) $$P_{c}(z)=e^{-\langle m\rangle }\sum_{m=1}^{\infty }\frac{{\langle m\rangle }^{m}}{\sigma m!\sqrt{2\pi m}}e^{-\frac{{\left(z-m\langle e_{1}\rangle /\tau \right)}^{2}}{2m\sigma^{2}}}.$$

Note that the function $P_{c}(z)$ was obtained within the simplified model of resistive transitions in the superconducting film which are triggered by absorption of individual photons. The accuracy of Equation (6) is limited due to assumption that statistical parameters of the single-photon charge distribution, $\langle e_{1}\rangle$ and $\sigma^{2}$, are the same regardless the number of absorbed photons per acquisition time. An example of the theoretical curve obtained after fitting an experimental histogram by Equation (6) is shown in Figure 2a by a magenta dashed line. Here and below, we make digital summation up to m=40. A fair agreement between experimental and fitting results can be obtained for most parts of experimental histograms. However, Equation (6) cannot adequately approximate wings of experimentally observed histograms P(z) at high z values. This is demonstrated in the inset to Figure 2a. Additionally, uncertainties of obtained distribution parameters are rather high (see Table 1). Nevertheless, the mean number of detected photo-counts 〈m〉 monotonically increases with the pump power, as should be expected.

Statistical distribution $P_{c}(z)$ does not take into account that parameters of the single current distribution in Equation (3) can be sensitive to a number of photons absorbed by a superconducting film. If the sensitivity is not very strong within considered levels of illumination, this effect can be accounted by replacing the constant single charge $\langle e_{1}\rangle$ with a linearly dependent charge $\langle e_{1}\rangle (1+Gm)$. In this case, Equation (6) transforms into

(7) $$P(z)=e^{-\langle m\rangle }\sum_{m=1}^{\infty }\frac{{\langle m\rangle }^{m}}{\sigma m!\sqrt{2\pi m}}e^{-\frac{{\left[z-m\langle e_{1}\rangle (1+Gm)/\tau \right]}^{2}}{2m\sigma^{2}}}$$

with an additional approximation parameter G. The solid curves in Figure 2 show results of approximation using Equation (7). It was checked for the histograms, recorded at a number of bolometer settings, that the improved distribution Equation (7) fits all parts of experimental histograms with sufficiently better accuracy than Equation (6). A positive sign of G values indicates that the single charge tends to increase when the radiation power grows up. The values of other fitting parameters for different histograms are presented in Table 1. Both the overall accuracy and individual uncertainties for most of the parameters are better than in the case of fitting by the simplified Equation (6).

**Figure 3:**
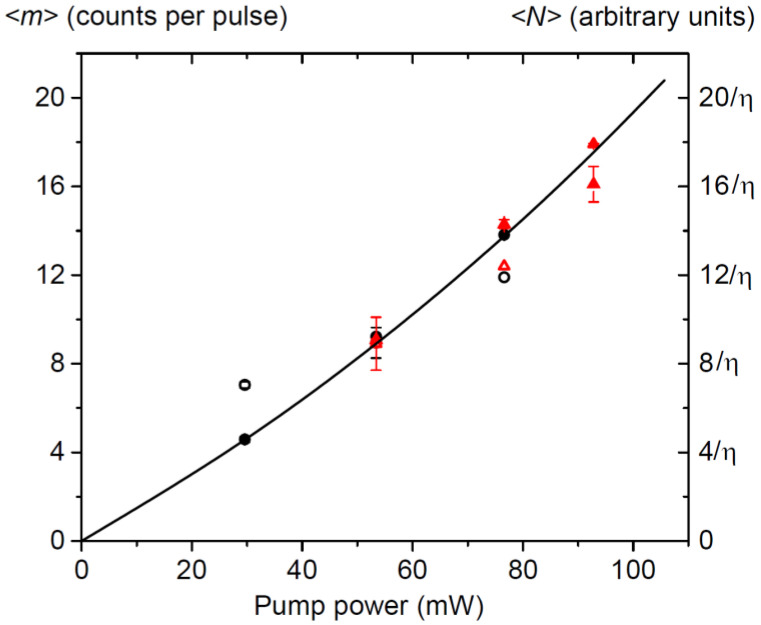
The mean number of the bolometer photo counts per pulse (in absolute unites, dots), and the theoretical prediction for the mean number of incident photons (in arbitrary units, a solid curve), both versus the time-averaged pump power. Open dots: results of approximation of experimental histograms by Equation (6), filled dots: by Equation (7). Black circular dots correspond to experimental data obtained under the HEB transport current of 64 µA, red triangular dots correspond to the data under the HEB transport current of 69 μA.

The bolometer transport current and voltage determine its operating point, on which the process of transition from the superconducting state of the bolometer to the normal one essentially depends. For this reason, the parameters of elementary current pulses can differ markedly. This is clearly seen in Table 1—the average values of single pulses for pump power values of 53.4 mW and 76.6 mW for a current of 69 μA are noticeably higher than for a current of 64 μA (at a fixed voltage $U_{b}=0.23\;V$). The variances of the mean values of single pulses also differ. This is what explains the difference between the corresponding curves in Figure 2. At the same time, as can be seen from Table 1 and from Figure 3, the average numbers of registered photo counts for the 2nd and 3rd graphs in Figure 2a and for the 1st and 2nd graphs in Figure 2b are in good agreement. It should also be noted that our formula for describing the distribution function of the bolometer photocurrent is a convolution of two functions, an asymmetric Poisson and symmetric Gaussian. In other words, the entire asymmetry of the graphs in Figure 2 is associated with the Poisson nature of the statistics of photo counts, which depends on their average number, but not with the operation mode of the bolometer. This is precisely what explains the success of obtaining the average number of photo counts, despite the difference in the graphs in Figure 2 in different operating modes of the bolometer.

For our study, 〈m〉 is the most important approximation parameter. In contrast to $\langle e_{1}\rangle$, $\sigma^{2}$, *G*, it should not depend on the bolometer parameters unless the quantum efficiency or an input radiation power changes. The true values of 〈m〉, hidden in experimental histograms, depend on the average number of incident photons. Figure 3 shows values of 〈m〉 determined using Equation (6) (open dots) and Equation (7) (filled dots) for histograms detected at various powers of PDC pump radiation. The solid curve shows theoretical dependence of the number of THz PDC photons on the pump power. It was calculated using strict Equations (1) and (2) with only one fitting parameter, which was the general scaling coefficient in Equation (1). It is seen that all results of approximation of experimental histograms follow this general dependency, no matter how other approximation parameters change. Deviation of points from the curve depends on the general uncertainty of approximation. The values, calculated using the probability distribution from Equation (7), correspond to behavior of the number of photons with better accuracy.

Therefore, application of the proposed probability distribution Equation (7) enables to reveal the discrete nature of detection by terahertz superconducting NbN bolometer. There exist a number of elementary detection events which finally originate the analog-type signal at the bolometer’s output. Since each individual event (count) can contribute to the output with very different amplitudes (single charges), the bolometer cannot operate in a strictly digital regime. However, now we can derive the general relation between the discrete photon statistics of the input radiation and the continuous probability density distribution P(z) of the analog output *z*.

To derive Equation (7) for the above approximations we accounted distribution of the number of single counts P(m) in the form of the Poisson function in Equation (5), which is applicable in the case of our radiation source. In general cases this distribution can take another form, depending on the statistics of the input photons P(N) [42],

(8) $$P(m)=\sum_{N=m}^{\infty }\frac{N!}{m!(N-m)!}\eta^{m}{\left(1-\eta \right)}^{N-m}P(N).$$

Thus, we can generalize Equation (7) for any type of discrete distribution P(N) by taking Equation (8) instead of Equation (5). The relationship between statistical distributions of the bolometer readings P(z) and of the number of input photons P(N) takes the form

(9) $$P(z)=\sum_{m=1}^{\infty }\frac{\eta^{m}}{m!\sqrt{2\pi m}\sigma }e^{-\frac{{\left[z-m\langle e_{1}\rangle (1+Gm)/\tau \right]}^{2}}{2m\sigma^{2}}}\sum_{N=m}^{\infty }\frac{N!}{(N-m)!}{\left(1-\eta \right)}^{N-m}P(N).$$

The obtained general expression allows one to determine relationship between statistical parameters of photons and analog bolometer readings, such as average values, dispersions, and different-order correlation functions. Its application opens a way for calibration of the bolometer parameters, including its quantum efficiency. In particular, this can be done in the future by measuring the moments of P(z) under bolometer irradiation by THz fields with specific quantum photon statistics.

## 4. Conclusions

In summary, we have measured and analyzed statistical distributions of the analog readings of an antenna-coupled THz bolometer with superconducting NbN film under a special type of irradiation by low-photon fluxes of THz photons with Poisson statistics and controllable average photon numbers. The photons were generated via low-gain parametric down-conversion in a nonlinear Mg:LiNbO_3_ crystal pumped by 10 ns 523 nm laser pulses. Together with the input elements and NbN film of each bolometer, the crystal was placed in a cooled He cryostat closed from thermal radiation from other external sources. It was shown that statistical distributions of bolometer readings per laser pulse can be approximated with high accuracy under the following assumptions:

(1) any reading consists of a number of discrete single counts with Poisson statistical distribution,
(2) contributions of individual counts (“single charges”) to each total reading are statistically distributed according to the normal law,
(3) the average values of contributions (“mean single charges”) depend on the number of counts in each reading.

By taking linear dependence of the mean single charge on the number of counts, Equation (7) is proposed to describe statistical distribution of the bolometer readings in case of Poisson statistics of the input photons. Parameters of the single charge distributions, such as dispersion, mean value of the single charge and its dependence on the number of detected counts, were obtained different for different bolometer transport currents. In direct accordance with the model, the mean numbers of bolometer counts, evaluated by fitting distributions at different bolometer settings, demonstrate a vanishingly weak dependence on these settings, but a high linear sensitivity to the number of input PDC photons. This result confirms the discrete nature of THz detection by superconducting bolometers and opens a way for studying their quantum characteristics. Finally, the general formula (Equation (9)) is proposed to describe the statistical distribution of analog bolometer readings under illumination by THz radiation with arbitrary photon statistics.

## Figures and Tables

**Table 1:** Results of approximation by two probability distributions, $P_{c}(z)$ from Equation (6) and P(z) from Equation (7). The uncertainties are the root-mean-square errors obtained under approximating the experimental dependences by the least squares method.

Bolometer Current	Pump Power	Number of Counts <*m*>		Mean Single Current <e_1_>/τ, arb.un.		Single-Current Deviation σ, arb.un.	
		Equation (6)	Equation (7)	Equation (6)	Equation (7)	Equation (6)	Equation (7)
64 μA	29.6 mW	7.04 ± 0.14	4.58 ± 0.12	22.9 ± 0.5	46.4 ± 1.9	44.9 ± 0.2	56.6 ± 1.0
	53.4 mW	8.95 ± 0.7	9.22 ± 0.06	43.8 ± 3.6	35.1 ± 0.4	37.7 ± 2.6	16.4 ± 0.5
	76.6 mW	11.9 ± 5.7	13.82 ± 0.03	45 ± 23	29.7 ± 0.15	35 ± 20	11.3 ± 0.2
69 μA	53.4 mW	8.9 ± 1.2	9.07 ± 0.06	80 ± 12	66.5 ± 0.5	66.8 ± 9.2	30.3 ± 1.2
	76.6 mW	12.4 ± 8.8	14.3 ± 0.2	71 ± 53	48.3 ± 1.0	54.7 ± 48.3	19.7 ± 1.6
	92.8 mW	17.91 ± 0.03	16.1 ± 0.8	87.9 ± 0.2	156 ± 4	19.7 ± 0.6	115 ± 4

## Data Availability

The data presented in this study is available in the article.
